# Cost-effectiveness analysis of human papillomavirus vaccination in South Africa accounting for human immunodeficiency virus prevalence

**DOI:** 10.1186/s12879-015-1295-z

**Published:** 2015-12-11

**Authors:** Xiao Li, Martinus P. Stander, Georges Van Kriekinge, Nadia Demarteau

**Affiliations:** Health Economics, GSK Vaccines, Avenue Fleming 20, 1300 Wavre, Belgium; Health Economic Research, HEXOR (Pty) Ltd, Block J, Central Park, 400 16th Road, Midrand, Republic of South Africa

**Keywords:** Cervical cancer, Vaccine, Human immunodeficiency virus, Human papillomavirus, South Africa, 2-dose, Cost-effective

## Abstract

**Background:**

This study aims at evaluating the cost-effectiveness of a 2-dose schedule human papillomavirus (HPV) vaccination programme of HPV and human immunodeficiency virus (HIV) naïve 12-year-old girls, in addition to cervical cancer (CC) screening alone, in South Africa. The study aims to account for both the impact of the vaccine among girls who are HIV-positive (HIV+) as well as HIV-negative (HIV-) population.

**Methods:**

A previously published Markov cohort model was adapted to assess the impact and cost-effectiveness of a HPV vaccination programme in girls aged 12 years (*N* = 527 900) using the AS04-adjuvanted HPV-16/18 vaccine from a public payer perspective. Two subpopulations were considered: HIV- and HIV+ women. Each population followed the HPV natural history with different transition probabilities. Model input data were obtained from the literature, local databases and Delphi panel. Costs and outcomes were discounted at 5 %. Extensive sensitivity analyses were conducted to assess the robustness of the evaluation.

**Results:**

Implementation of the AS04-adjuvanted HPV-16/18 vaccine in combination with current cytological screening in South African girls could prevent up to 8 869 CC cases and 5 436 CC deaths over the lifetime of a single cohort. Without discounting, this HPV vaccine is dominant over screening alone; with discounting, the incremental cost-effectiveness ratio is ZAR 81 978 (South African Rand) per quality-adjusted life years (QALY) gained. HPV vaccination can be considered cost-effective based on World Health Organization (WHO) recommended threshold (3 x gross domestic product/capita = ZAR 200 293). In a scenario with a hypothetical targeted vaccination in a HIV+ subpopulation alone, the modelled outcomes suggest that HPV vaccination is still cost-effective, although the incremental cost-effectiveness ratio increases to ZAR 102 479. Results were sensitive to discount rate, vaccine efficacy, HIV incidence and mortality rates, and HPV-related disease transition probabilities.

**Conclusions:**

The AS04-adjuvanted HPV-16/18 vaccine can be considered cost-effective in a South African context although the cost-effectiveness is expected to be lower in the HIV+ subpopulation than in the overall female population. With improved access to HIV treatment, the HIV mortality and incidence rates are likely to be reduced, which could improve cost-effectiveness of the vaccination programme in South Africa.

**Electronic supplementary material:**

The online version of this article (doi:10.1186/s12879-015-1295-z) contains supplementary material, which is available to authorized users.

## Background

Human papillomavirus (HPV) is the necessary cause of cervical cancer (CC) [1], with an estimated 528 000 annual incident CC cases and 266 000 CC deaths worldwide in 2012 [2]. High-risk regions, with age-standardised incidence over 30/100 000 women, include Eastern Africa (42.7/100 000), Melanesia (33.3/100 000), Southern Africa (31.5/100 000) and Middle Africa (30.6/100 000) [2]. Most frequent HPV types found in CC are types HPV-16 and HPV-18 which account for approximatively 70 % of all cases worldwide [3, 4]. Many of the regions with high CC incidence, such as South Africa, are also burdened with high incidence of human immunodeficiency virus (HIV) infection [5, 6]. The high CC incidence could be partially related to the high HIV incidence. Individuals infected with HIV have an increased risk of HPV infection and the associated subsequent disease as a result of impaired immunity [7, 8]. A strong association between HIV-positive (HIV+) status and the prevalence of oncogenic HPV types, specifically HPV-16 and HPV-18, has been previously documented [7, 9, 10].

Three HPV vaccines are available throughout the world: the AS04-adjuvanted HPV-16/18 vaccine (*Cervarix*^®^, GSK), the HPV-6/11/16/18 vaccine (*Gardasil*, Merck & Co., Inc.) and the HPV-6/11/16/18/31/33/45/52/58 vaccine (*Gardasil 9*, Merck & Co., Inc.). These vaccines can be used for the prevention of HPV-related diseases such as CC. Efficacy of these vaccines has been demonstrated in adolescents and adult women [11–15]. Moreover, HPV vaccines clinical trials in HIV-infected individuals have shown that these vaccines have clinically acceptable safety profiles and are immunogenic [16, 17].

Up to mid-2014, more than 50 countries worldwide introduced vaccination against HPV in their national immunisation programme for girls [18] including South Africa [19]. It has been reported that more countries are preparing to offer pre-adolescent girls HPV vaccination, including many African countries where a high HIV prevalence exists [20].

A systematic review on the cost-effectiveness, clinical impact and health economic impact of HPV vaccination concluded that routine vaccination of girls is cost-effective compared with CC screening alone [21]. One South African study reported an incremental cost-effectiveness ratio (ICER) per quality-adjusted life-year (QALY) gained of 1 078 US dollar (USD) from a health service perspective [22]. However, to our knowledge, no model explicitly has taken into account the specificity of a HIV infected subpopulation in South Africa. Given the specific epidemiology of HPV infection and associated disease among this specific population, accounting for these in health economic evaluations in countries with a high prevalence of HIV may influence the cost-effectiveness of the implementation of a HPV vaccination programme. Also of interest would be to assess the effect of current change in HIV treatment and epidemiology on the cost-effectiveness of the HPV vaccination.

This study aims to assess the cost-effectiveness from a public payer perspective of a 2-dose AS04-adjuvanted HPV-16/18 vaccination programme added to the current CC screening programme, compared with the current CC screening programme (Papanicolaou smear test; Pap) alone in South Africa accounting for both the impact of the vaccine among the HIV+ as well as the HIV- population.

## Methods

### Model description

**Fig. 1:**
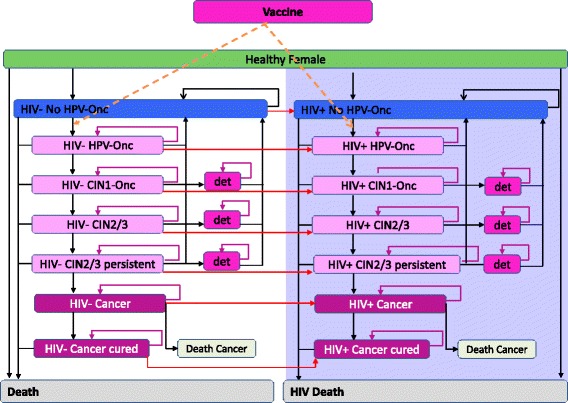
Model structure. The model assesses the impact and cost-effectiveness of a HPV vaccination on HIV- and HIV+ subpopulations, considering the HPV natural history with different transition probabilities. The left panel shows the natural history of HPV infection, with overlay of CC screening and vaccination. At any time during the cohort’s life, subjects can move from the left panel (HPV natural history, HIV-) to the right panel (HPV natural history, HIV+). CIN, cervical intraepithelial neoplasia; CIN1, CIN grade 1; CIN2/3, CIN grade 2 or 3; CIN1-Onc, oncogenic HPV types caused CIN1; det, subjects with disease detected through screening (same pathways but different probabilities), HIV, human immunodeficiency virus; HIV-, HIV-negative; HIV+, HIV-positive; HPV, human papillomavirus; Pap, Papanicolaou smear test; HPV-Onc, oncogenic HPV types

A previously published lifetime Markov process cohort decision tree with a one year cycle length focusing on oncogenic HPV was adapted to account for both HIV+ and HIV- subpopulations [23]. The model consisted of a series of health states representing the natural history of HPV infection and CC (see Fig. 1 for details) as in the initial model. The initial model was however duplicated to reproduce HPV natural history in both the HIV+ and HIV- subpopulations. Subjects moved between different health states over annual cycles throughout the disease process, governed by transition probabilities specific for each health state. Throughout the model, a proportion of the population yearly acquired HIV at an age-specific incidence rate reported for South Africa hence moving to the HIV+ sub-model.

### Population

A 12-year-old girls’ cohort entered the model and was followed over lifetime. It was assumed that this cohort was both HIV- and HPV-naïve at the start of the simulation (i.e. mother-to-child HIV transmission was not included in the model). Based on 2011 mid-year population estimates in South Africa, a cohort of 527 900 girls aged 12 years was considered for this analysis [24].

### Model input data and assumptions

#### Natural history related to oncogenic HPV

Different transition probabilities were applied to the HIV-positive and negative populations to account for the specificities of the acquisition and natural history of HPV infection in these two subpopulations, as documented by a recent systematic literature review [25]. Age-dependent HIV incidence was taken into account to estimate the proportion of the population moving from the HIV- to HIV+ over the lifetime of the cohort. The transition probabilities related to the natural history of oncogenic HPV among HIV- subpopulation remained unchanged compared with Suarez et al. [23]. Those related to HIV+ subpopulation were consolidated from the findings of a targeted literature review via PubMed using search terms related to HPV, HIV, cervix and neoplasm. Articles published before February 2014 were retrieved and the abstracts and selected manuscripts were assessed by a single reviewer. When data for HIV+ subjects were unavailable in the literature, conservative assumptions were made (i.e. same cervical intraepithelial neoplasia [CIN] treatment success rate in HIV+ and HIV- subpopulation). See Additional file 1. 

The model was calibrated on incidence and mortality of CC in the HIV- population in South Africa by adjusting the transition probability from persistent CIN2/3 to CC as this parameter could not be retrieved from the literature due to the lack of these data for ethical reasons. For the HIV+ population, we assumed a multiplication factor of 2 for this specific transition probability, based on a prospective cohort study conducted by Massad et al. on progression and regression rates for abnormal cervical cytology among HIV-infected women [26]. The study reported greater progression rate within 6 months after diagnosis among HIV+ when compared with HIV- women (14 % vs. 7 %). See Table 1 for details on all annual transition probabilities included in the model.

**Table 1:** Natural history of oncogenic HPV in HIV- and HIV+ subpopulations input data

Natural history-related parameter	Annual probability			
	HIV- subpopulation	Source	HIV+ subpopulation	Source
HPV-Onc to CIN1	0.049	Adjusted from [60] (0.15 after 36 months and 0.21 after 60 months)	0.096	[49]
HPV-Onc to CIN2/3	0	Spontaneous progression from HPV-onc to CIN2/3 within 1 year; assumed to be 0	0	Assumption (same as HIV- subpopulation)
HPV-Onc clearance to normal	0.293–0.553	[29, 60–62]	0.212	[49]
CIN1 to CIN2/3	0.091	[61–63]	0.098	[49]
CIN2/3 cured	0.227	[61–63]	0.227	Assumption (same as HIV- subpopulation)
CIN2/3 to CIN1	0	Spontaneous regression from CIN2/3 to CIN1 within 1 year; assumed to be 0; all patients cured going to no HPV assumption	0	Assumption (same as HIV- subpopulation)
HIV- CIN2/3 to persistent CIN2/3	0.114	[62, 63]	0.114	Assumption (same as HIV- subpopulation)
Persistent CIN2/3 to cervical cancer	Age 15: 0.00 %	Based on estimated CIN2/3 and reported cervical cancer progression rates	Age 15: 0.00 %	Applied relative risk ratio of 2 for HIV+ based on the progression rate from LSIL to HSIL [64]
	Age 35: 1.10 %		Age 35: 2.20 %	
	Age 45: 2.52 %		Age 45: 5.04 %	
	Age 60: 7.56 %^a^		Age 60: 15.12 %^a^	

^a^The model calibration led to 0.12 % yearly increase from 20 years of age to 35 years, 0.2 % yearly increase from 36 years of age to 45 years, 0.3 % yearly increase from 45 years of age to 55 years, and 0.4 % yearly increase from 60 years of age onwards

CIN, cervical intraepithelial neoplasia; CIN1, CIN grade 1; CIN2/3, CIN grade 2 or 3; HPV, human papillomavirus; HPV-Onc, oncogenic HPV types; HIV, human immunodeficiency virus; HIV-, HIV-negative; HIV+, HIV + positive; HSIL, high-grade squamous intraepithelial lesions; LSIL, low-grade squamous intraepithelial lesions

#### Country specific data

Parameters related to HPV and HIV disease-specific incidence, background mortality rate, disease management, including CC screening and treatment of precancerous lesions and cancer were adapted to the South African setting and retrieved from the literature, local databases or expert opinion (see Table 2).

**Table 2:** HPV and HIV epidemiology and disease management input data

Parameter	Annual probability			
	HIV- subpopulation	Source	HIV+ subpopulation	Source
Mortality rate				
General mortality rate	Age 15: 0.09 %	[65]	Age 15: 0.10 %	[65]
	Age 30: 0.20 %		Age 30: 4.62 %	
	Age 45: 0.51 %		Age 45: 7.71 %	
	Age 60: 1.56 %		Age 60: 29.72 %	
	Age 75: 5.31 %		Age 75: 37.38 %	
HPV and HIV incidence				
HIV incidence rate in women	NA	-	Age 15: 0.025	[65]
			Age 30: 0.004	
			Age 45: 0.001	
			Age 60: 0.001	
			65+ years: 0	
Oncogenic HPV incidence	0.041–0.390	[59]	0.110–1.000	[59, 66]
Cervical cancer parameters				
Cervical cancer mortality	0.110	[2]	0.291	[67]
Cervical cancer cured	0.151	[2]	0.027	[67]
HPV screening-related parameters				
Screening coverage	13.6 %	[59]	13.6 %	[59]
Screening ages	30–60 years	[63]	30–60 years	[63]
Screening frequency	Every 3 years	WHO [27, 28]	Every 3 years	WHO [27, 28]
CIN1 detected	0.58	[68]	0.58	[68]
CIN2/3 detected	0.61	[68]	0.61	[68]
HPV-related disease management				
Proportion of women treated if CIN1 is detected	0.50	Assumption	0.50	Assumption
CIN1 treatment success	0.90	Assumption	0.90	Assumption
Proportion of women treated if CIN2/3 is detected	1	Assumption	1	Assumption
CIN2/3 treatment success	0.90	Assumption	0.90	Assumption

ASSA, Actuarial Society of South Africa; CIN, cervical intraepithelial neoplasia; CIN1, CIN grade 1; CIN2/3, CIN grade 2 or 3; HIV, human immunodeficiency virus; HIV-, HIV-negative; HIV+, HIV-positive; HPV, human papillomavirus; ICO, Institut Català d'Oncologia; Pap, Papanicolaou smear test

#### Cervical cancer screening

South Africa CC screening guidelines recommend that each woman from the age of 30 should be screened every 10 years at no cost [27]. The start age for CC screening was set to 30 years of age and at a frequency of once every 3 years until 60 years of age, as the South Africa national guideline and World Health Organization (WHO) recommend this frequency as best case for CC prevention or when resources allow for it [27, 28]. A cytological screening (i.e., Papanicolaou smear test) was assumed and sensitivity for the detection of cervical abnormalities with this test was retrieved from a meta-analysis on this subject. See Table 2 for details.

#### Utilities

Utilities for precancerous lesions, CC and CC survivors in South Africa were not available from the literature. Disutility values used were therefore retrieved from published reports for other settings and assumed to be identical for HIV- and HIV+ subpopulations (see Table 3) [29–33]. Baseline utility values in the absence of HPV disease for HIV- and HIV+ subpopulations were assumed to be 1 and 0.81, respectively [34].

**Table 3:** Utility input data [29–33]

Health state	Disutility value	Utility value (HIV-); baseline value = 1	Utility value (HIV+); baseline value = 0.81
No HPV	0	1	0.81
HPV-Onc	0	1	0.81
CIN1	0	1	0.81
CIN1 det	0.013	0.987	0.797
CIN2/3	0	1	0.81
CIN2/3 det	0.013	0.987	0.797
Cervical cancer	0.273	0.727	0.537
Cervical cancer cured	0.062	0.938	0.748

CIN, cervical intraepithelial neoplasia; CIN1, CIN grade 1; CIN2/3, CIN grade 2 or 3; det, detected; HIV, human immunodeficiency virus; HIV-, HIV-negative; HIV+, HIV + positive; HPV, human papillomavirus

#### Resource utilisation and disease-related costs

Only direct medical costs were considered in this analysis. In accordance with the South Africa Pharmacoeconomic Guidelines, direct non-healthcare-related-costs and indirect costs associated with quality-of-life and productivity loss were not included [35].

A two-round Delphi panel with 8 South African experts in the field of gynaecologic oncology was conducted to collect medical management-related resource use for patients with HPV-related disease in South Africa. The overall resources used for the treatment of precancerous lesions and CC included the resources associated with general practitioner (GP)/specialist visits, diagnostic procedures, treatments and hospitalisations. The reference unit costs (year 2013) for each of these identified resources were obtained from the South Africa Uniform Patient Fee Schedule (UPFS) [35] or from the literature when not available in the database (see Additional file 2 for details). Weighted averages of resource use multiplied with unit costs were included as cost input data into the model (Table 4). All costs are expressed in South African Rand (ZAR). Cost data extracted from the literature and reported in international dollar were converted to same year ZAR values using the Power-Purchase-Parity (PPP) adjusted exchange rate between ZAR and USD for the value year in the publication [36, 37]. These estimated same year ZAR values were then inflation-adjusted to 2013 using the South African Consumer Price Index data [38].

**Table 4:** Costs input data [36–38]

Intervention	Costs (ZAR)
Cost of regular screening negative Pap smear	256
Regular screening + false positive	256 + 1 656 (assume 2 % false positive)
Treatment CIN1 detected	830
Treatment CIN2/3 detected	2 464
Cervical cancer stage I-IV	40 507

CIN, cervical intraepithelial neoplasia; CIN1, CIN grade 1; CIN2/3, CIN grade 2 or 3; Pap, Papanicolaou smear test

#### Vaccine costs

The 2013 list price of the AS04-adjuvanted HPV-16/18 vaccine of ZAR 595.39 per dose was used as the cost of vaccine per dose [39]. The national tender price is likely to be lower than its listed price; however, the tender price is unknown. A 2-dose schedule was assumed following the recent approval of the 2-dose schedule in South Africa.

#### Vaccine effectiveness and coverage

A proxy vaccine effectiveness was used, based on the most recent data on the AS04-adjuvanted HPV-16/18 vaccine efficacy reported for each endpoint irrespective of the causative type in the lesion in the Total Vaccinated Cohort from the end-of-study results from the PATRICIA trial [15]. Reported vaccine efficacy was used as a proxy for effectiveness as follows: CIN1+ 50.3 % (95 % CI: 40.2–58.8 %; CIN1 health state in the model), CIN2+ 64.9 % (95 % CI: 52.7–74.2 %; CIN2/3 health state) and CIN3+ 93.2 % (95 % CI: 78.9–98.7 %; CC health state) [15]. Vaccine efficacy in HIV+ subjects is assumed to be the same as those reported for HIV- cohort in the PATRICIA trial, based on recently reported immunogenicity results from a phase I/II clinical trial in HIV+ women in South Africa showing the vaccine is immunogenic and does not impact on HIV disease progression in HIV+ women [16].

A vaccination schedule with 2 doses for the target age-group was assumed, as recommended by the WHO recently [40], and approved by the South African health authorities. Vaccine efficacy for the 2-dose schedule was assumed identical to the vaccine efficacy reported for a 3-dose schedule based on the result of recent immunogenicity studies demonstrating non-inferiority of a 2-dose vs. a 3-dose schedule [41–43]. In the base case analysis, we assume 100 % vaccination coverage (which does not impact the cost-effectiveness of the vaccine as the model used has a static design) and lifetime vaccine-induced protection in both HIV- and HIV+ subpopulations based on a mathematical modelling prediction [44].

#### Discounting

In accordance with South Africa Pharmacoeconomic Guidelines for economic evaluations, future costs and outcomes were discounted at a rate of 5 % [35].

#### Data availability

All input data were retrieved from publicly available sources and access to the ASSA2008 model was granted by the actuarial society of South Africa in 2012 and confirmed in 2015.

### Model outcomes

#### Base case analysis

For the base case analysis, a 2-dose AS04-adjuvanted HPV-16/18 vaccine in combination with the current Pap smear screening programme, compared with the current Pap smear screening programme alone, was assessed. Discounted and undiscounted cost, health outcomes, incremental values and cost-effectiveness ratios were estimated.

According to the WHO recommendation for pharmacoeconomic evaluations, an intervention can be considered cost-effective when the ICER is below the threshold of 3 × gross domestic product (GDP) per capita [45] and highly cost-effective below 1 x GDP per capita. GDP per capita at current prices in South Africa was ZAR 66 764 in 2013 [36].

#### Scenario analyses

The cost-effectiveness of the AS04-adjuvanted HPV-16/18 vaccine in a strictly hypothetical HIV+ population was specifically modelled as for the base case analysis. When assessing the HIV+ subpopulation, the HPV infection rate in the HIV- population was set to be 0, to restrict the estimation of HPV related burden in the HIV+ population. The vaccination cost was attributed to girls that would become HIV+ over their lifetime. The subject would acquire HIV according to the South African incident rate and would be included in the HIV+ subpopulation. This analysis corresponds to a sub-evaluation of girls of whom the future HIV status would be known at the time of vaccination. Discounted and undiscounted outcomes were investigated.

#### Sensitivity analysis

- ➢ Overall
- To account for the uncertainty in model parameters, a one-way sensitivity analysis on the discounted ICER was performed, varying key parameters in the model by ±20 % (x0.8 – x1.2) from their baseline values where 95 % confidence interval (CI) was not available. For vaccine efficacy, the 95 % CI values were taken as minimum and maximum values (see Additional file 3).
- ➢ HIV+ subpopulation
- Although the vaccine efficacy and duration of protection remains unknown in HIV+ subpopulation, we have evaluated the impact of changes by means of one-way sensitivity analysis on the discounted ICER, by varying the vaccine effectiveness and vaccine duration of protection using the following values:
  - vaccine effectiveness: relative reduction of 5 % (×0.95) to 30 % (×0.70) with a 5 % increment of the base case value
  - vaccine duration of protection: waning of immunity from 5 to 50 years after initial vaccination, followed by a booster dose for 40 % of the cohort at the time of waning.
- ➢ Vaccine price
- A vaccine price sensitivity analysis on the discounted ICER varying the vaccine price with a range of 50 to 150 % of the list price per dose was performed.

#### Two-way sensitivity analysis

Impact of changes in HIV mortality and HIV incidence rate on the discounted ICER were evaluated using a two-way sensitivity analysis. The mortality was reduced by 10 % (×0.90) to 50 % (×0.50) and HIV incidence was varied by ±75 % (×0.25 – ×1.75). The HIV mortality under which the vaccine becomes highly cost-effective among the overall population was estimated by determining the reduction in HIV mortality under which the ICER reached 1 × GDP per capita (highly cost-effective).

#### Probabilistic sensitivity analysis

A probabilistic sensitivity analysis (PSA) was performed for both base case and scenario analyses using *@Risk* (Palisade Corporation, Ithaca, New York, USA) to test parameter uncertainty and to evaluate the overall robustness of the model. Distributions were assigned to transition probabilities, costs and utility using normal distribution (limited from 0 to 1 for probabilities) where confidence intervals were available, otherwise, uniform distribution was assigned ranging from 20 % above and below the base case value (see Additional file 4). In total, 10 000 samples were generated from the assigned distribution.

## Results

### Model validation

**Fig. 2:**
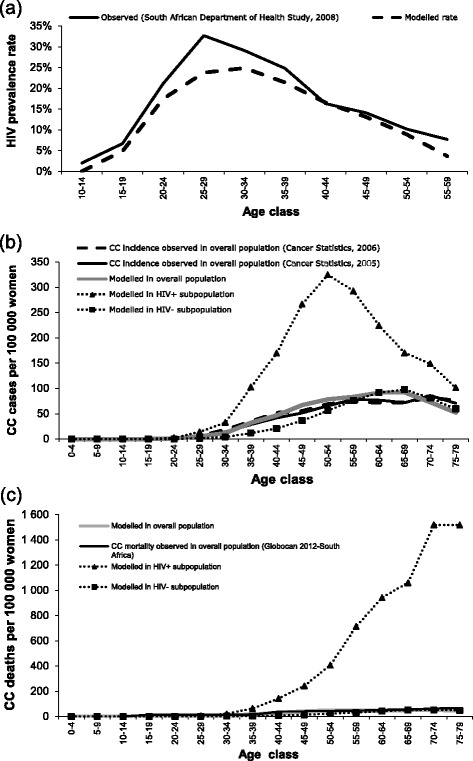
Modelled vs. observed: (**a**) HIV prevalence, (**b**) cervical cancer incidence, (**c**) cervical cancer mortality. Sources: HIV prevalence [57]; cervical cancer incidence [58], cervical cancer mortality [59]. CC, cervical cancer; HIV, human immunodeficiency virus; HIV+, HIV-positive; HIV-, HIV-negative

The Markov cohort model adequately reproduced the age-dependent HIV prevalence rate, CC incidence and CC mortality rate for South Africa, as reported in Fig. 2. The modelled CC incidence and mortality rate in the HIV+ subpopulation was significantly higher compared with the HIV- subpopulation. This could however not be validated against observed data as these do not exist.

### Base case analyses

Undiscounted and discounted costs and outcomes comparing CC screening in addition to HPV vaccination with CC screening alone for a single cohort of 12-year-old girls in South Africa are reported in Table 5. By implementing HPV vaccination, 8 869 (undiscounted) CC cases and 5 436 CC deaths (undiscounted) could be prevented over the lifetime of a single cohort of girls in South Africa. Total incremental undiscounted cost was ZAR −663 748 191 and 68 270 QALY were gained. The undiscounted results thus show that the HPV vaccination programme added to the current CC screening programme is dominant over the CC screening programme alone. Discounted results reported were ZAR 471 064 695 overall cost difference and 5 746 QALY gained, resulting in an ICER of 81 978 ZAR/QALY. Thus, HPV vaccination with the AS04-adjuvanted HPV-16/18 vaccine can be considered to be cost-effective according to the WHO recommendations [36, 45].

**Table 5:** Base case results: overall 12-years-old girls cohort

Outcomes	Screening	Screening + HPV vaccination	Incremental value	ICER (ZAR/QALY)
NO DISCOUNTING				
Total costs	ZAR 1 649 427 899	ZAR 985 679 708	ZAR −663 748 191	
Vaccine cost	ZAR 0	ZAR 628 612 762	ZAR 628 612 762	
Screening cost	ZAR 149 297 117	ZAR 153 547 788	ZAR 4 250 671	
CIN1 treatment cost	ZAR 10 577 161	ZAR 3 746 430	ZAR −6 830 731	
CIN2/3 treatment cost	ZAR 8 262 222	ZAR 1 813 136	ZAR −6 449 086	
CC treatment cost	ZAR 1 481 291 399	ZAR 197 959 592	ZAR −1 283 331 807	
Life years	27 173 430	27 230 436	57 006	
CC cases	10 244	1 376	−8 869	
CC deaths	6 313	877	−5 436	
QALYs	26 554 764	26 623 034	68 270	V + S dominant
DISCOUNTED AT 5 %				
Total costs	ZAR 221 807 577	ZAR 692 872 271	ZAR 471 064 695	
Vaccine cost	ZAR 0	ZAR 628 612 762	ZAR 628 612 762	
Screening cost	ZAR 35 277 421	ZAR 36 453 101	ZAR 1 175 681	
CIN1 treatment cost	ZAR 2 775 840	ZAR 1 007 252	ZAR −1 768 588	
CIN2/3 treatment cost	ZAR 2 541 718	ZAR 553 386	ZAR −1 988 332	
CC treatment cost	ZAR 181 212 598	ZAR 26 245 770	ZAR −1 988 332	
Life years	1 283 817	9 154 639	4 633	
QALYs	8 904 494	8 910 240	5 746	ZAR 81 978

CC, cervical cancer; CIN, cervical intraepithelial neoplasia; CIN1, CIN grade 1; CIN2/3, CIN grade 2 or 3; QALYs, quality-adjusted life-years; ZAR, South African Rand; HPV, human papillomavirus; ICER, incremental cost-effectiveness ratio; V + S, HPV vaccination + screening

### Scenario analysis

In the scenario analysis specific for the HIV+ subpopulation, a total of 1 813 CC cases (undiscounted) and 1 707 CC deaths (both undiscounted) could be prevented over the lifetime of the cohort. Total undiscounted cost difference with CC screening alone resulted in ZAR −46 418 189 and a QALY gain of 7 248 for a single cohort of girls aged 12 in South Africa, thus resulting in the HPV vaccination programme added to the current cervical screening programme to be dominant over the CC screening programme alone in a HIV+ subpopulation. Discounted at 5 %, results reported were ZAR 136 358 673 overall cost difference and 1 331 QALY gained, resulting in an ICER of 102 479. Thus, HPV vaccination with the AS04-adjuvanted HPV-16/18 vaccine can be considered to be cost-effective according to the WHO recommendations in this specific subpopulation [36, 45] (Table 6).

**Table 6:** Scenario results: HIV+ subpopulation

Outcomes	Screening	Screening + HPV vaccination	Incremental value	ICER (ZAR/QALY)
NO DISCOUNTING				
Total costs	ZAR 295 251 951	ZAR 248 833 762	ZAR −46 418 189	
Vaccine cost	ZAR 0	ZAR 183 228 152	ZAR 183 228 152	
Screening cost	ZAR 19 794 328	ZAR 21 262 010	ZAR 1 467 682	
CIN1 treatment cost	ZAR 3 641 306	ZAR 557 658	ZAR −3 083 648	
CIN2/3 treatment cost	ZAR 3 545 580	ZAR 561 971	ZAR −2 983 609	
CC treatment cost	ZAR 268 270 736	ZAR 43 223 970	ZAR −225 046 766	
Life years	3 179 736	3 186 707	6 971	
CC cases	2 161	348	−1 813	
CC cancer deaths	2 035	328	−1 707	
QALYs	2 573 677	2 580 925	7 248	V + S dominant
DISCOUNTED AT 5 %				
Total costs	ZAR 62 169 185	ZAR 198 527 858	ZAR 136 358 673	
Vaccine cost	ZAR 0	ZAR 183 228 152	ZAR 183 228 152	
Screening cost	ZAR 5 766 054	ZAR 6 223 334	ZAR 457 279	
CIN1 treatment cost	ZAR 1 126 206	ZAR 177 452	ZAR −948 755	
CIN2/3 treatment cost	ZAR 1 183 601	ZAR 190 676	ZAR −992 925	
CC treatment cost	ZAR 54 093 324	ZAR 8 708 245	ZAR −45 385 079	
Life years	1 283 817	1 285 076	1 259	
QALYs	1 039 522	1 040 852	1 331	ZAR 102 479

CC, cervical cancer; CIN, cervical intraepithelial neoplasia; CIN1, CIN grade 1; CIN2/3, CIN grade 2 or 3; QALYs, quality-adjusted life-years, ZAR, South African Rand; HPV, human papillomavirus, ICER, incremental cost-effectiveness ratio; V + S, HPV vaccination + screening

### Sensitivity analyses

**Fig. 3:**
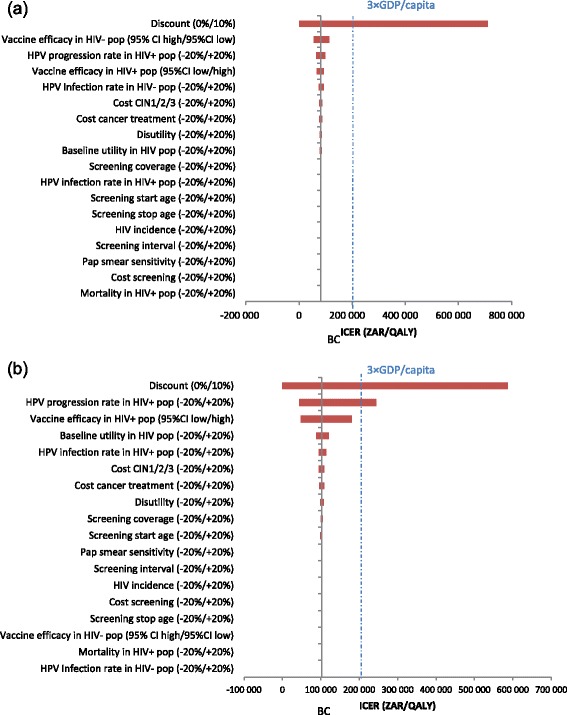
One-way sensitivity analyses: (**a**) base case analysis, (**b**) scenario analysis. BC, base case; CI, confidence interval; CIN, cervical intraepithelial neoplasia; CIN1/2/3, CIN grade 1, 2 or 3; GDP, gross domestic product; HIV, human immunodeficiency virus; HIV-, HIV-negative; HIV+, HIV-positive; HPV, human papillomavirus; ICER, incremental cost-effectiveness ratio; Pap, Papanicolaou test; pop, population

One-way sensitivity analyses on variation of key variables and the resulting ICER are reported in Fig. 3, showing that variation in the discount rate had the greatest impact on ICER estimates. For the overall population base case analysis (Fig. 3a), vaccine efficacy in HIV- subpopulation, HPV progression rate and vaccine efficacy in HIV+ subpopulation had a significant impact on the ICER. In the sensitivity analysis specific to the HIV+ subpopulation (Fig. 3b), main drivers of ICER estimates were HPV progression rate, vaccine efficacy and baseline utility in the HIV+ subpopulation.

**Fig. 4:**
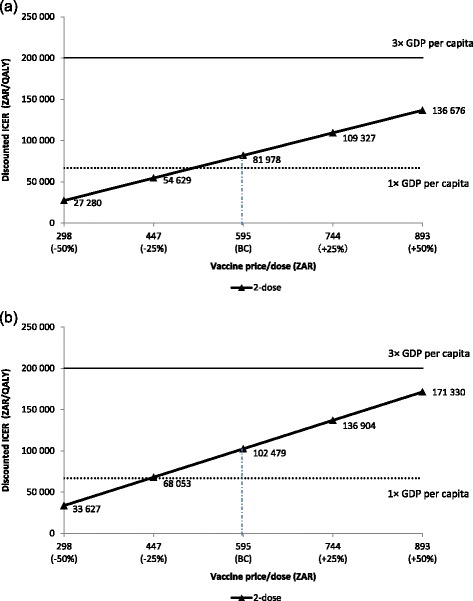
One-way price sensitivity analysis: (**a**) base case analysis, (**b**) scenario analysis. BC, base case; GDP, gross domestic product; HIV, human immunodeficiency virus; HIV+, HIV-positive; ICER, incremental cost-effectiveness ratio; ZAR, South African Rand

In the overall population analysis, with 50 % increase in the vaccination price (Fig. 4a), results show that HPV vaccination is still cost-effective. When the vaccine price is reduced by 25 %, HPV vaccination becomes highly cost-effective in South Africa. In the HIV+ subpopulation analysis, the price range between highly cost-effective and cost-effective vaccination price is between ZAR 450 and 950 per vaccine dose respectively (Fig. 4b).

**Fig. 5:**
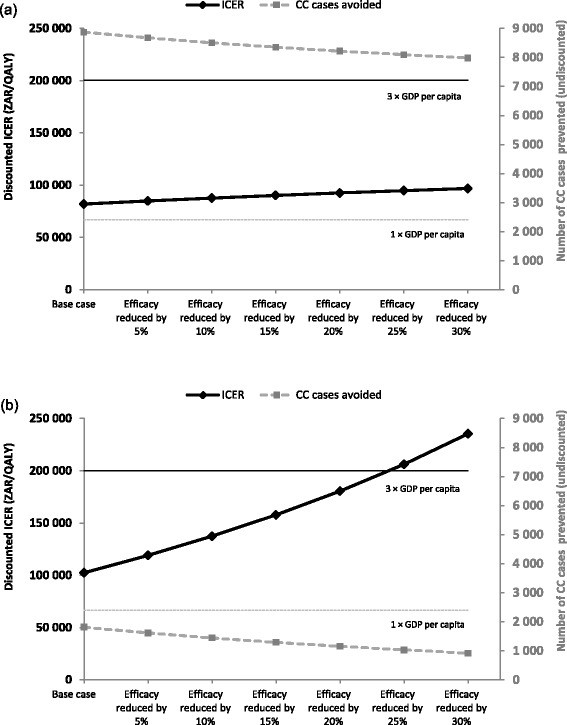
Sensitivity analyses: impact of vaccine efficacy: (**a**) base case analysis, (**b**) scenario analysis. CC, cervical cancer; GDP, gross domestic product; ICER, incremental cost-effectiveness ratio; ZAR, South African Rand

**Fig. 6:**
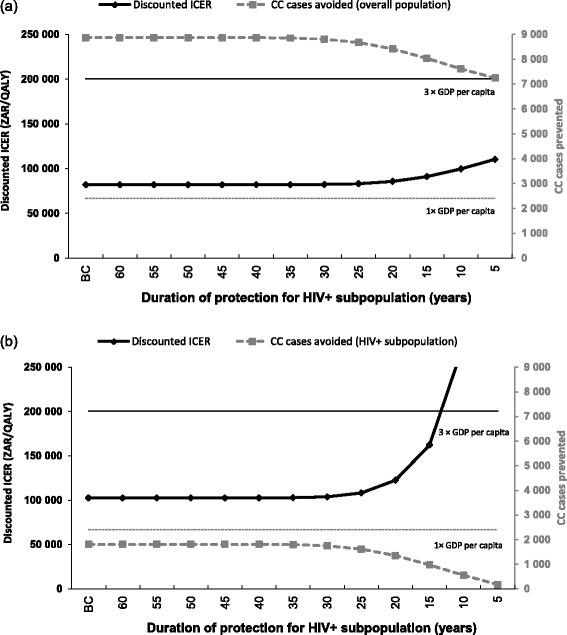
Impact of duration of protection in HIV+ subpopulation: (**a**) overall population, (**b**) HIV+ subpopulation. BC, base case; CC, cervical cancer; GDP, gross domestic product; HIV, human immunodeficiency virus; HIV+, HIV-positive; HIV-, HIV-negative; ICER, incremental cost-effectiveness ratio; ZAR, South African Rand

When the impact of vaccine efficacy (Fig. 5) on ICER estimates was assessed, results show that HPV vaccination remained cost-effective in the overall population when vaccine efficacy was reduced by 30 %. However, in the HIV+ subpopulation analysis, HPV vaccination became not cost-effective if vaccine efficacy was reduced by 25 %. A similar trend was observed for the impact on the duration of protection in the HIV+ subpopulation (Fig. 6) with the discounted ICER remaining below the cost-effectiveness threshold in the overall population but rapidly rose above the threshold if the duration of protection was shorter than 15 years in the HIV+ subpopulation.

The two-way sensitivity analysis reported in Table 7 demonstrates that with changes in HIV incidence (−75 to +75 %) and HIV mortality rate (from no change to −50 %) vaccination remained cost-effective or highly cost-effective in all scenarios in the overall population.

**Table 7:** Two way sensitivity analysis: mortality and incidence rates in HIV+ subpopulation

Incidence	HIV+ mortality (base case)	HIV+ mortality −10 %	HIV+ mortality −20 %	HIV+ mortality −30 %	HIV+ mortality −40 %	HIV+ mortality −50 %
NO DISCOUNTING						
−75 %	V + S dominant	V + S dominant	V + S dominant	V + S dominant	V + S dominant	V + S dominant
−50 %	V + S dominant	V + S dominant	V + S dominant	V + S dominant	V + S dominant	V + S dominant
−25 %	V + S dominant	V + S dominant	V + S dominant	V + S dominant	V + S dominant	V + S dominant
Base case	V + S dominant	V + S dominant	V + S dominant	V + S dominant	V + S dominant	V + S dominant
25 %	V + S dominant	V + S dominant	V + S dominant	V + S dominant	V + S dominant	V + S dominant
50 %	V + S dominant	V + S dominant	V + S dominant	V + S dominant	V + S dominant	V + S dominant
75 %	V + S dominant	V + S dominant	V + S dominant	V + S dominant	V + S dominant	V + S dominant
DISCOUNTED AT 5 %						
−75 %	*85 537*	*84 256*	*82 724*	*80 862*	*78 551*	*75 609*
−50 %	*83 958*	*81 600*	*78 845*	*75 590*	*71 693*	*66 948*
−25 %	*82 798*	*79 513*	*75 754*	*71 421*	**66 383**	**60 466**
Base case	*81 978*	*77 880*	*73 275*	*68 080*	**62 189**	**55 473**
25 %	*81 435*	*76 610*	*71 279*	**65 375**	**58 824**	**51 540**
50 %	*81 116*	*75 635*	*69 667*	**63 167**	**56 089**	**48 385**
75 %	*80 979*	*74 896*	*68 362*	**61 352**	**53 842**	**45 817**

The bold zone indicates the ICER is < 1 × GDP per capita (ZAR 66 764); V + S, HPV vaccination + screening

**Fig. 7:**
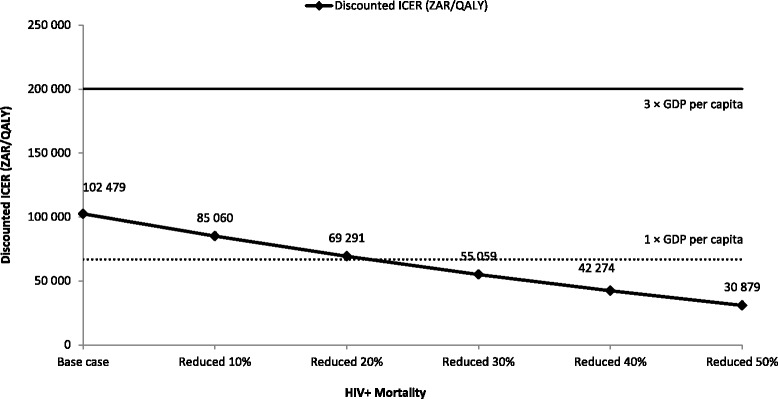
Sensitivity analyses: impact of HIV mortality rate in the HIV+ subpopulation. CC, cervical cancer; GDP, gross domestic product; HIV, human immunodeficiency virus; HIV+, HIV-positive; ICER, incremental cost-effectiveness ratio; QALY, quality-adjusted life years; ZAR, South African Rand

In the HIV+ subpopulation, a 22 % decrease in the HIV mortality rate, at current HIV incidence, lead to HPV vaccination becoming highly cost-effective (< 1 × GDP per capita) (Fig. 7).

**Fig. 8:**
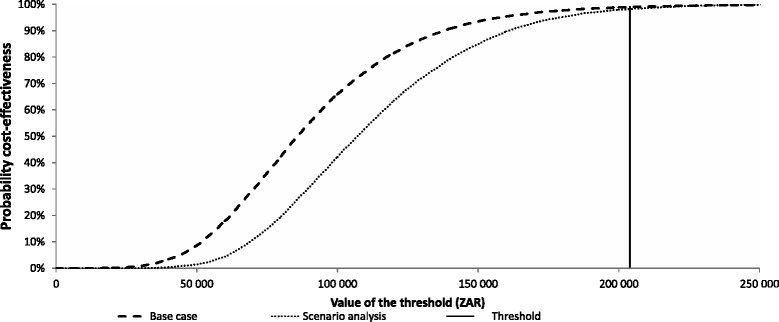
Cost-effectiveness acceptability curves for the base case and scenario analysis. ZAR, South African Rand

The results of the probabilistic sensitivity analysis are reported in the acceptability curve in (Fig. 8), which presents the cumulative probability in function of discounted ICER analysis. The probability of ICER being below the threshold (3 × GDP per capita) is 99 % for base case and 98 % for scenario. It shows that the scenario analysis has a slower slope towards the maximum indicating that the spread of the samples has a larger interval than for the base case. This may be due to more parameters for the scenario analysis to have been subjected to uncertainty than for the base case.

The results indicate that even under parameter variation, HPV vaccination in South Africa is likely to be cost-effective in all target groups showing the important public health impact of vaccination in this setting.

## Discussion

We evaluated the cost-effectiveness of a CC prevention programme in South Africa where HPV vaccination is added to the current CC screening programme. In this evaluation, we explicitly accounted for the existing HIV+ population in South Africa that have specific HPV-related disease history and hence a potential impact on the value of a HPV programme. To our knowledge, no analysis of this kind has been previously reported. The base case analysis (including 29 % HIV+ and 71 % HIV- over the lifetime of a single cohort of 12-year-old South African girls) showed that this strategy would be cost-effective when compared with CC screening alone. Similar conclusions have been previously reported in other settings although these analyses did not include HIV+ subpopulations [22, 23, 29, 46, 47].

Evidence exists demonstrating that the acquisition and progression of HPV is faster in HIV+ individuals making them more at risk of HPV-related cancers [7, 20, 48, 49]. Different transition probabilities were therefore applied to the HIV- positive and negative populations to account for the specificities of the acquisition and natural history of HPV infection in these two subpopulations, as documented by a recent systematic literature review [25]. Moreover, there is no sufficient evidence in the literature that HIV treatments have effect on the HPV natural history [50]. Vaccination would likely be the only way to effectively prevent the increased risk of HPV and HPV-related outcomes in a HIV+ population.

In this modelling exercise, HPV vaccination was considered for a cohort of HPV and HIV naïve South African 12-year-old girls. These girls may become HPV and/or HIV positive post vaccination. A specific scenario analysis on HIV+ population was conducted. This represents the population target at the age of vaccination as at that age we assumed no existing HIV infection. The modelled outcomes suggest that HPV vaccination remained cost-effective under the WHO recommended cost-effectiveness threshold in the overall population as well as in the HIV+ subpopulation, although the ICER (ZAR 102 479 per QALY gained) is higher than the one estimated for the overall population (ZAR 81 978 per QALY gained). Decrease in overall HIV- associated mortality rate for the HIV+ population would make HPV vaccination more attractive as more disease can be avoided in later life. Given current mortality rates in HIV+ women, most HIV+ women may die before they develop precancerous lesions, or indeed cancer, even when HPV disease develops faster in HIV+ individuals. With better access to HIV treatment in South Africa, HIV incidence and mortality rate is however likely to improve in the near future, and hence improve the cost-effectiveness of the vaccination programme. Results were most sensitive to variation of discount rate, vaccine efficacy, duration of vaccine-induced protection in the HIV+ subpopulation, HIV incidence and mortality rates and HPV vaccine price. The parameter with the largest impact on the ICER was the discount rate. A high discount rate favours disease preventive interventions that are close to the time of disease onset. For CC specifically, a high discount rate disfavours HPV vaccination as CC take years or even decades to develop [51, 52]. A recent update of the National Institute for Health and Care Excellence (NICE) in the United Kingdom recommends a discount rate of 1.5 % to be used in technological appraisals for diseases with an expected benefit leading to prevention of death or a benefit that is sustained for at least 30 years [53]. Applying this discount rate would favour prevention of disease and death further away in the future and improve cost-effectiveness for HPV vaccination substantially (results not shown).

A systematic literature review on the cost-effectiveness of HPV vaccination in middle and low income countries, including South Africa [54], found that most studies in these countries concluded that HPV vaccination is likely to be cost-effective or possibly even cost saving. An evaluation in the South African context specifically concluded that adding HPV vaccination can be considered highly cost-effective [22]. Our study estimated a higher ICER in the base case evaluation than the one reported in this previous evaluation (ZAR 102 479 ~ USD 10 609 vs. USD 1 078 per QALY gained) from a public payers perspective. Besides the inclusion of HIV-specific health states in the current evaluation, the ICER difference between the two evaluations was driven by a lower 3 % discount rate used in Sinanovic et al. evaluation compared with the 5 % in the present evaluation. The Sinanovic et al. study was indeed published before the issue of the National Pharmacoeconomics Guideline mandating a 5 % discount rate as baseline [35]. Cytological screening was used in both studies as screening strategy in South Africa. Sinanovic et al. used 3 times screening at 10-years interval starting at 30 years of age [22]. A screening with 3 years interval from age 30 to 60 years was used in this analysis, as recommended by WHO and the national guideline, if resources allow [27, 28]. A Pap test was assumed, an HPV DNA testing, with higher sensitivity and more automated process, may be recommended as triage testing or replace the current screening in the future which would also impact the results. The treatment costs used in our study were based on a Delphi panel resulting in lower costs than included in the Sinanovic study which used updated costs from two earlier publications [37, 55]. Finally, the vaccine cost also differed. While the Sinanovic et al. study assumed vaccination cost of USD 570 including 3 doses and a booster rate of 50 %, administration, wastage and vaccination programmatic costs, we however applied the vaccine price with the 2-dose schedule (ZAR 595.39 using listed price) based on the recent regulatory update.

Another population-level modelling study assessed the economic impact of the different CC prevention (screening and vaccination) in Sub-Saharan African countries. It demonstrated that HPV vaccination alone is highly cost-effective in South Africa when price per course is USD 100 (ICER = USD 4 900 per life year saved). The authors also acknowledge the need of including HIV status in future modelling [56].

The tender price is likely to be lower than the listed price, which would further improve the cost-effectiveness of the vaccine compared with no vaccination. However under variation of the vaccine price (from 50 % decrease to 50 % increase), HPV vaccination remained cost-effective. Therefore higher vaccination cost including e.g. administration costs or wastage would still result in the vaccine to be cost-effective in South Africa. In this analysis, all scenarios used the cost of a 2-dose vaccination schedule, reducing the price of the vaccine, compared with a 3-dose HPV vaccination schedule. Furthermore, using a 2-dose schedule may alleviate not only issues related to overall vaccination budget (also including e.g. administration, vaccine storage, transportation, …) but additionally increase acceptability and hence compliance of the vaccine. These elements were not accounted for in this presented analysis [41].

Limitations of this analysis exist. The Markov model used here is a static model and therefore no dynamic effects such as herd protection were taken into account. Only the direct vaccine effect was considered which provides a conservative estimation of the effect of vaccination. Furthermore, adaptation of a dynamic model would require detailed inputs data for both HIV+ and HIV- population which would be very challenging to obtain and, hence, requiring many assumptions to be made which could drive the results of the analysis and add uncertainties. The use of a static model does also add transparency to both the analysis and data selection. The difference in HIV prevalence rate from age 20 to 45 between the model and the observed data (see Fig. 2) may be due to observed data being reported based on an annual cross-sectional population survey while the model follows a single age-cohort over lifetime based on today’s epidemiology and HIV treatment practice.

Most of the country-specific data in South Africa are reported at the population level (including both HIV+ and HIV- subpopulations). It is therefore very challenging to extract different parameters for either subpopulation from the reported data. Therefore, several conservative assumptions had to be made with regards to parameters for disease progression or CC mortality rate. Also, comparison with HIV+ specific CC incidence was not possible as such data are not reported anywhere. Furthermore, children born with HIV were not included in this analysis. Since the model follows HPV disease progression in both HIV-infected and uninfected population, this model implicitly assumes that HIV was acquired by sexual transmission.

Our analysis also did not address inequities in healthcare access that may exist in and between populations in South Africa. Access to CC screening may not be readily available for all women in South Africa leading to excess risk of this cancer in unscreened women [29]. HPV vaccination given to girls age 12 may, to a certain extent, improve access to prevention of CC for large groups in the population thereby creating more opportunities for individual development and generation of well-being. Moreover, there is not sufficient evidence that treatment of HIV has effect on HPV natural history in the literature; vaccination would likely be the only way to effectively alleviate the increased risk of HPV and HPV-related outcomes in HIV+ girls.

Although studies using mathematical simulation modelling cannot replace clinical trial-based evaluation, model-based analyses provide important information that can help prioritise and guide the implementation of healthcare choices in South Africa.

However, this analysis highlights that potential reduction in the future incidence of HIV in a population may impact the cost-effectiveness value of an HPV vaccine by lowering the ICER value*.* Also, this analysis highlights that reduction in HIV mortality, which can be expected with new treatments, would also improve the cost-effectiveness of the vaccination. This should be taken into account when implementing a vaccination programme among girls that for some will develop HIV and hence could benefit more or benefit less of the programme depending on the future of HIV treatments or epidemiology.

## Conclusions

Findings from this modelling exercise suggest that the introduction of the 2-dose AS04-adjuvanted HPV-16/18 vaccine in South Africa added to the current CC screening programme may lead to a strong reduction in the number of CC cases and related deaths in the HIV+ as well as the HIV- subpopulations and may be a cost-effective intervention in both the general overall female population and the HIV+ female subpopulation alike. Improvement in HIV treatment leading to better survival among the HIV+ further improved the cost-effectiveness of the implementation of a HPV vaccination programme in countries with high HIV prevalence such as South Africa.

## Endnotes

*Cervarix*^®^ is a registered trade mark of the GSK group of companies.

## Additional files

Additional file 1:
**Literature Review: natural history related to oncogenic HPV in HIV infected population in Africa or South Africa.** (PDF 167 kb) Additional file 2:
**Resource use unit costs and cervical cancer treatment costs.** (PDF 328 kb) Additional file 3:
**Input ranges for one way sensitivity analyses.** (PDF 156 kb) Additional file 4:
**Multivariate probabilistic sensitivity analysis input data.** (PDF 64 kb)

## Abbreviations

ASSA: Actuarial Society of South Africa
BC: base case
CC: cervical cancer
CI: confidence interval
CIN: cervical intraepithelial neoplasia
CIN1: cervical intraepithelial neoplasia grade 1
CIN2/3: cervical intraepithelial neoplasia grade 2 or 3
CPI: consumer price index
det: detected subjects with disease detected through screening
EX: exchange rate
GDP: gross domestic product
GP: general practitioner
HIV: human immunodeficiency
HIV-: human immunodeficiency virus-negative
HIV+: human immunodeficiency virus-positive
HPV: human papillomavirus
HPV-Onc: oncogenic HPV types
HSIL: high-grade squamous intraepithelial lesions
ICER: incremental cost-effectiveness ratio
ICO: Institut Català d’Oncologia
IMF: International Monetary Fund
INT$: international dollar
LSIL: low-grade squamous intraepithelial lesions
NICE: National Institute for Health and Care Excellence
Pap: Papanicolaou smear test
Pop: population
PPP: purchasing power parity
QALY: quality-adjusted life years
UPFS: Uniform Patient Fee Schedule
USD: US dollar
V + S: vaccination plus screening
WHO: World Health Organization
ZAR: South African Rand
