# Supplementary material for: Cost-effectiveness analysis of human papillomavirus vaccination in South Africa accounting for human immunodeficiency virus prevalence
Source: BMC Infect Dis. 2015 Dec 11;15:566. doi: 10.1186/s12879-015-1295-z (PMC4676856; doi:10.1186/s12879-015-1295-z)
Supplement: Additional file 1: — Literature Review: natural history related to oncogenic HPV in HIV infected population in Africa or South Africa. (PDF 167 kb) [file 12879_2015_1295_MOESM1_ESM.pdf]

Additional file 1 - Literature Review: natural history related to oncogenic HPV in HIV infected population in Africa or South Africa

Supplementary Table 1: PICOS framework

The study question was formalised according to the PICOS framework (Population, Intervention, Comparison, Outcome and Study type) to capture studies reporting natural history related to oncogenic HPV in HIV infected population in Africa or South Africa.

Population	HIV infected women in South Africa or subsaharan Africa or Africa
Intervention	None
Comparator	None
Outcome	Progression, regression rate, cervical cancer prevalence, incidence and mortality rate
Study design	No restriction

Database: Pubmed
Time span: 2000-2014/2/17
Language: English only

Search keywords

#1	Human papillomavirus OR HPV OR cervix OR neoplasm
#2	Human immunodeficiency virus infection OR acquired immune deficiency syndrome OR HIV
#3	progression OR regression OR prevalence OR incidence OR mortality
#4	Africa OR South Africa OR subsaharan Africa
#5	#1 AND #2 AND #3 AND #4 AND #5

Total number of hits 821

Exclusion criteria

- Not written in English
- Not a country of interest
- Not the study type of interest (e.g. case report, randomised controlled clinical trials),
- Non-human studies
- Men population
- Not numerical rate reported

From: Moher D, Liberati A, Tetzlaff J, Altman DG, The PRISMA Group (2009). *P*referred *R*eporting *I*tems for *S*ystematic Reviews and *M*eta-*A*nalyses: The PRISMA Statement. PLoS Med 6(6): e1000097. doi:10.1371/journal.pmed1000097

For more information, visit www.prisma-statement.org

Supplementary Figure 1: PRISMA 2009 Flow Diagram

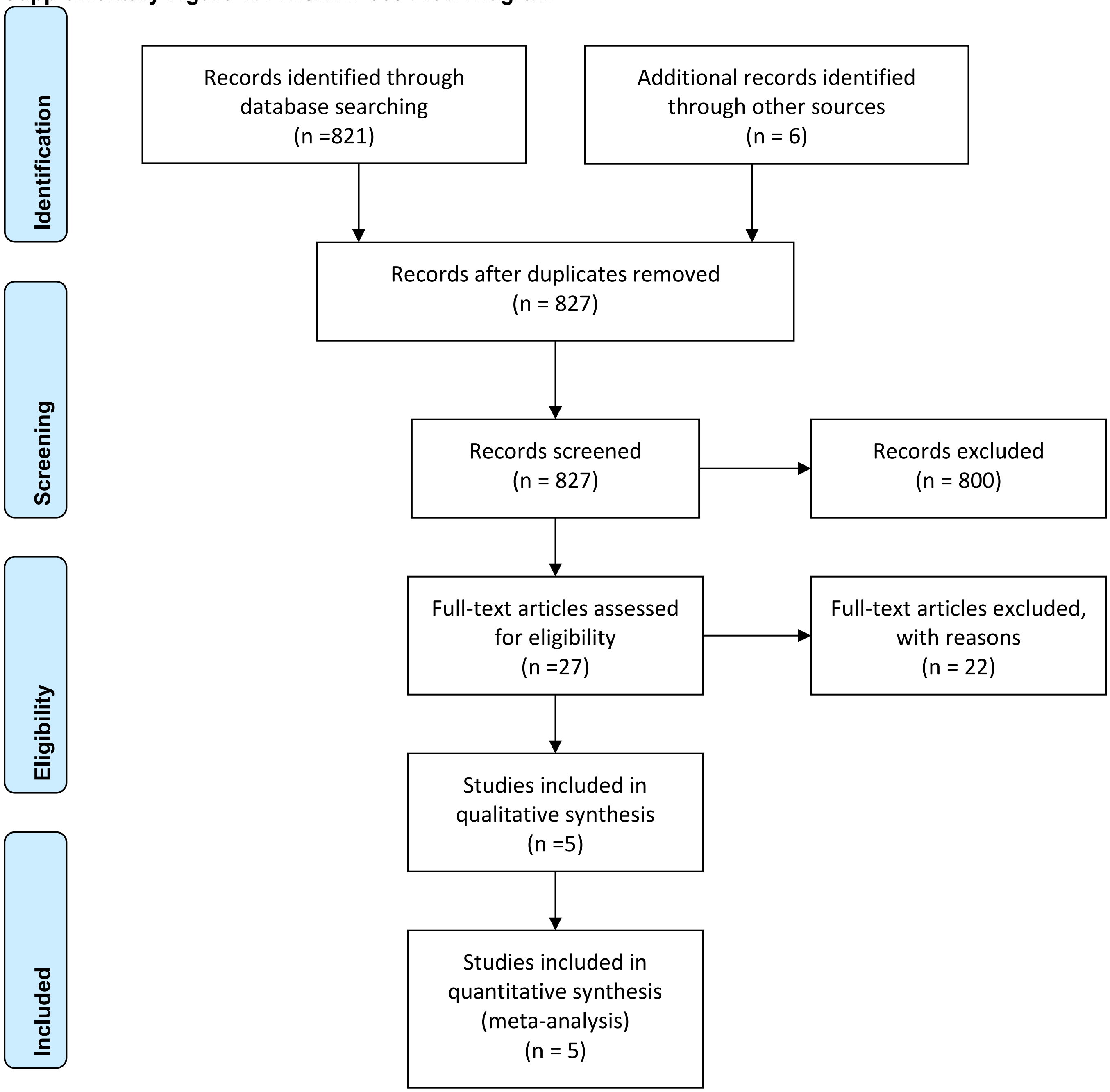

From: Moher D, Liberati A, Tetzlaff J, Altman DG, The PRISMA Group (2009). *P*referred *R*eporting *I*tems for *S*ystematic Reviews and *M*eta-*A*nalyses: The PRISMA Statement. PLoS Med 6(6): e1000097. doi:10.1371/journal.pmed1000097

For more information, visit www.prisma-statement.org
