# Supplementary material for: Cost-effectiveness analysis of human papillomavirus vaccination in South Africa accounting for human immunodeficiency virus prevalence
Source: BMC Infect Dis. 2015 Dec 11;15:566. doi: 10.1186/s12879-015-1295-z (PMC4676856; doi:10.1186/s12879-015-1295-z)
Supplement: Additional file 2: — Resource use unit costs and cervical cancer treatment costs. (PDF 328 kb) [file 12879_2015_1295_MOESM2_ESM.pdf]

Additional file 2 – Resource use unit costs and cervical cancer treatment costs

Supplementary Table 1: Unit costs

Items	Unit cost (ZAR)	Year	Cost adjustment (Cost*CPI*Exchange rate) (ZAR)	Source
Cost of visit				
Anaesthetist	1 520.00	2013	NA	UPFS 2013 [1]
Dermatologist	183.00	2013	NA	UPFS 2013 [1]
GP	79.00	2013	NA	UPFS 2013 [1]
Gynaecologist	183.00	2013	NA	UPFS 2013 [1]
HIV physician	183.00	2013	NA	UPFS 2013 [1]
Infectious disease specialist	183.00	2013	NA	UPFS 2013 [1]
Oncologist	183.00	2013	NA	UPFS 2013 [1]
Psychologist	183.00	2013	NA	UPFS 2013 [1]
Radiologist	183.00	2013	NA	UPFS 2013 [1]
Cost of diagnostic procedures				
Chest X ray	197.00	2013	NA	UPFS 2013 Code DA0520 [2]
HIV test	47.80	2013	NA	NHLS 2013 code 877 [3]
HPV tests	857.00	2013	NA	NHLS 2013 code 3920 [3]
Pap smears	57.90	1999	57.90*1*2.2=125.00	Goldie et al. [4]; CPI: Statistical office [5]
Pregnancy test	73.50	2013	NA	NHLS 2013 code 3692
Repeat Pap smear	57.90	1999	57.90*1*2.2=125.00	Goldie et al. [4]
Ultrasound	799.00	2013	NA	UPFS 2013 Code DA0530[2]
Hospitalisation cost				
In-patient, normal room	1 384.00	2013	NA	UPFS 2013[2]
In-patient, intensive care unit	3 499.00	2013	NA	UPFS 2013[2]
Day care	884.00	2013	NA	UPFS 2013 [2]
Treatment cost				
Hysterectomy	INT$ 1 582.70	2000	INT$1 582.7*3.12* 2.1=10 141.00	Goldie et al. [4]; Exchange rate (PPP) IMF [6]; CPI: Statistical office [5]
Laser treatment	INT$ 378.60	2000	INT$378.60*3.12*2.1=2 426.00	Assumption: Same cost as LLETZ, Goldie et al. [4]; EX(PPP) IMF [6] ; CPI: Statistical office [5]
LLETZ	INT$ 378.60	2000	INT$378.6*3.12*2.1=2 426.00	Goldie et al. [4]; EX(PPP) IMF [6] ; CPI: Statistical office [5]
Colposcopies	123.10	1999	123.1*2.2=226.00	Goldie et al. [4]; CPI: Statistical office [5]
Conisation	246.20	1999	246.2*2.2 =553.00	Goldie et al. [4]; CPI: Statistical office [5]
Biopsy	187.20	1999	187.2*2.2=405.00	Goldie et al. [4]; CPI: Statistical office [5]
Cryotherapy	652.70	1999	652.6*2.2=1 412.00	Goldie et al. [4] ; CPI: Statistical office [5]

CPI, consumer price index; EX, exchange rate; GP, general practitioner; HPV, human papillomavirus; HIV, human immunodeficiency; IMF, International Monetary Fund; INT$, international dollar; LLETZ, large loop excision of the transformation zone; NA, not applicable; NHLS, National Health Laboratory Service; Pap, Papanicolaou smear test; PPP, purchasing power parity; UPFS, Uniform Patient Fee Schedule ; ZAR, South African Rand.

Supplementary Table 2: Cervical cancer treatment costs

Items	Unit cost (ZAR)	Year	Number of cycles	Source
Cost of radiotherapy				
Planning facility fee	1 291	2013	1	UPFS 2013 [1]
Planning professional fee	553	2013	1	UPFS 2013 [1]
Radiation	17 025	2013	5	UPFS 2013 [1]
Radiation	7 295	2013	5	UPFS 2013 [1]
Remote afterloading	4 167	2013	1	UPFS 2013 [1]
Remote afterloading	9 779	2013	1	UPFS 2013 [1]
Sum	***ZAR 40 110***			
Cost of chemotherapy				
Facility fee	3 205	2013	5	UPFS 2013 [1]
Professional fee	6 470	2013	5	UPFS 2013 [1]
Sum	***ZAR 9 675***			
Cost of colostomy for rectovaginal fistulae				
Consultation specialist	183	2013	NA	UPFS 2013 [1]
Anaesthetic	1 520	2013	NA	UPFS 2013 [1]
Ward fee 5 days	3 660	2013	NA	UPFS 2013 [1]
Major theatre procedure: facility fee	2 420	2013	NA	UPFS 2013 [1]
Theatre procedure specialist	391	2013	NA	UPFS 2013 [1]
Sum	***ZAR 8 174***			

NA, not applicable; ZAR, South African Rand.

Delphi panel

A two-round Delphi panel was held over the course of 2012 and 2013.

In the first round, 8 panellists were selected to participate into the Delphi panel, using a standardised questionnaire querying 4 domains:

- Section 1: the description of panelists' specific expertise
- Section 2: the medical management of patients undergoing a cervical screening in South Africa
- Section 3: the medical management of CIN1, CIN2 and CIN3 and cervical cancer treatment
- Section 4: the medical management for genital warts treatment

As we did not include genital warts into our analysis, we don't report the data collected on genital warts treatment.

The questionnaire is attached (see below Delphi panel questionnaire).
The 8 panellists were all gynaecology oncologists. The regions covered by the panelists are: Gauteng, Western Cape Metro east, Western Eastern Cape Namibia, entire KZN, Tshwane and Johannesburg.

Results on the number of patients seen per year by each expert and the number of patients seen per year for cervical cancer screening is reported in Supplementary Figure 1.

Supplementary Figure 1: Number of patients in practice of experts

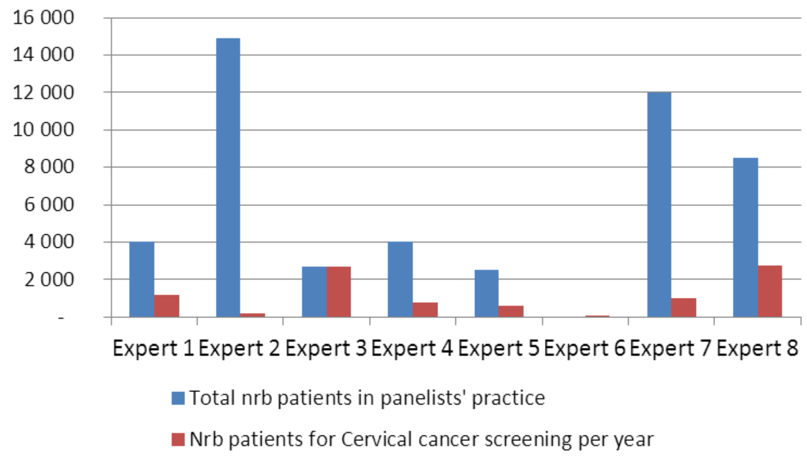

Supplementary Figure 2: Number of patients seen per year for treatment of CIN and cervical cancer

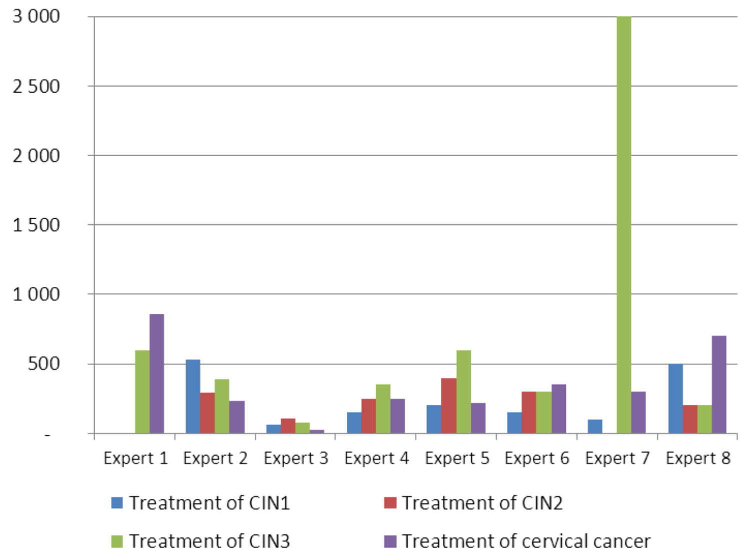

CIN 1/2/3, cervical intraepithelial neoplasia grade 1/2/3

Supplementary Table 3: Resource and cost items for negative pap smear

Number of times consumed	Resource	Proportion of patients receiving service	Unit cost (ZAR)	Weighted cost (ZAR)
1	Papanicolaou smear test	100%	125.29	125.3
1	GP visit	50%	79	39.5
1	Gynaecologist visit	50%	183	91.5
**Total weighted cost**		**ZAR 256**		

GP, general practitioner; ZAR, South African Rand.

Supplementary Table 4: Resource use and cost items for positive Pap smear

Number of times consumed	Resource	Proportion of patients receiving service	Delphi panel results	Unit cost (ZAR)	Weighted cost (ZAR)
	No additional procedure	22%	Majority agreed	0.0	0
2	Colposcopies	49%	All Agreed	266.4	261.0
1	Colposcopies with biopsy	30%	All Agreed	671.5	201.4
1.2	Repeat Pap smears	43%	All agreed	125.3	64.6
1	HPV tests	11%	Majority agreed	857.2	94.3
1	Ultrasound	6%	Majority agreed	799.0	47.9
1	HIV test	15%	All agreed	47.8	7.2
1	LLETZ	40%	Majority agreed	2 425.9	970.4
1	Pregnancy test	5%	All agreed	73.5	3.7
1	Chest X ray	3%	Majority agreed	197.0	5.9
**Total weighted cost**		**ZAR 1 656**			

HPV, human papillomavirus; HIV, human immunodeficiency ; LLETZ, large loop excision of the transformation zone; Pap, Papanicolaou smear test; ZAR, South African Rand.

Supplementary Table 5: Resource use and cost items for CIN1

Number of times consumed	Resource	Proportion of patients receiving service	Delphi panel results	Unit cost (ZAR)	Weighted cost (ZAR)
Medical management of histology confirmed CIN1: physician visit					
1.4	GP visits per patient	34%	Majority agreed	79	37.6
1.2	Gynaecologist visits per patient	44%	Majority agreed	183	96.6
1	Other (Infectious disease specialist)	17%	All Agreed	183	31.1
1	Other (oncology)	5%	Majority agreed	183	9.2
1.4	Pap smear	72%	Majority agreed	183	184.5
0	Days in hospital (in-patient, normal room)	0%	Majority agreed	1 384	0.0
0	Days in hospital (in-patient, intensive care unit)	0%	All agreed	3 499	0.0
0.2	Days in hospital (day care)	2%	Majority agreed	884	3.5
Total weighted cost		***ZAR 362.5***			
Medical management of histology confirmed CIN1: treatment procedure					
	No treatment procedure	85%	Estimated average	0	0.0
1	Laser treatment	4%	Estimated average	2 425.88	84.9
1	Cryotherapy	1%	Estimated average	1 412.27	14.1
1	Conisation	17%	Estimated average	532.75	90.6
1	Hysterectomy	2%	Estimated average	10 140.53	152.1
1	Other (Repeat Pap smear)	100%	Majority agreed	125.29	125.3
Total weighted cost		***ZAR 467.0***			
**Total for CIN1**		**ZAR 830**			

CIN1, cervical intraneoplasia grade 1; GP, general practitioner; Pap smear, Papanicolaou smear test; ZAR, South African Rand.

Supplementary Table 6: Resource use and cost items for CIN2/3

Number of times consumed	Resource	Proportion of patients receiving service	Delphi panel results	Unit cost (ZAR)	Weighted cost (ZAR)
Medical management of histology confirmed CIN2/3: Physician visits					
1.4	GP visits per patient	21%	Majority agreed	79	23.2
2.2	Gynaecologist visits per patient	56%	Majority agreed	183	225.5
1	Other (Infectious disease specialist)	21.20%	All agreed	183	38.8
1	Other (oncologist)	5%	All agreed	183	9.2
1	Other (HIV physician)	11%	Majority agreed	183	20.1
1.6	Pap smear per patient	65%	All agreed	183	190.3

1.4	Days in hospital (in-patient, normal room)	0%	Majority agreed	125.29	0.0
0	Days in hospital (in-patient, intensive care unit)	0%	All agreed	3499	0.0
0.27	Number of days in hospital (day care)	26%	Estimated average	884	60.5
Total weighted cost		***ZAR 567.6***			
Medical management of histology confirmed CIN2/3: treatment procedure					
	None	12%	Majority agreed	0	0.0
0	Laser treatment	0%	Majority agreed	2 425.88	0.0
1	Cryotherapy	18%	Majority agreed	1 412.27	254.2
1	Conisation	66%	Majority agreed	1 412.27	932.1
1	Hysterectomy	7%	All agreed	10 140.53	709.8
Total weighted cost		***ZAR 1 896.1***			
**Total for CIN2/3**		**ZAR 2 464**			

CIN2/3, cervical intraneoplasia grade 2/3; GP, general practitioner; HIV, human immunodeficiency; Pap smear, Papanicolaou smear test; ZAR, South African Rand.

Supplementary Table 7: Resource use and cost items for cervical cancer

Number of times consumed	Resource	Proportion of patients receiving service	Delphi panel results	Unit cost (ZAR)	Weighted cost (ZAR)
Medical management of histology confirmed Cervical Cancer: Physician visits					
3	GP visits per patient	59%	All agreed	79	139.8
3	Gynaecologist visits per patient	70%	Majority agreed	183	384.3
4	Oncologist visits per patient	54%	Majority agreed	183	395.3
2	Infectious disease specialist	22%	All agreed	183	80.5
4	Psychologist / care worker	45%	Majority agreed	183	329.4
1	HIV physician	2%	Majority agreed	183	3.7
1	Anaesthetist visits per patient	10%	Majority agreed	1 520	152.0
2	Radiologist visits per patient	27%	Majority agreed	183	98.8
1	Pap smears per patient	55%	Majority agreed	125.29	68.9
Total weighted cost		***ZAR 1 653***			
Medical management of histology confirmed cervical cancer: hospitalisation					
7	Days in hospital (in-patient, normal room)	27%	All agreed	1 384	2 615.76
2	Days in hospital (in-patient, intensive care unit)	5%	Majority agreed	3 499	349.9
1	Days in hospital (day care)	25%	Majority agreed	884	221
Total weighted cost		***ZAR 3 186.7***			
Medical management of histology confirmed cervical cancer: treatment procedure					
	No treatment procedure	3%	All agreed	0	0.0
0	Laser treatment	0%	All agreed	2 425.88	0.0
0	Cryotherapy	0%	All agreed	1 412.27	0.0
1	Conisation	8%	Majority agreed	532.75	42.6
	Hysterectomy	22%	All agreed	10 140.53	2 230.9
	Radiotherapy	75%	All agreed		
	25 sessions external beam	75%		40 110.0	30 082.5
	5 sessions brachytherapy				0.0
	Chemotherapy - 5 cycles platinum	30%	Estimated average	9 675.0	2902.5
	Brickers urinary diversion	3%	Majority agreed	8 174.0	204.4
	Colostomy for rectovaginal fistulae	3%	Majority agreed	8 174.0	204.4
Total weighted cost		***ZAR 35 667.3***			
**Total for cervical cancer**		**ZAR 40 507**			

GP, general practitioner; HIV, human immunodeficiency virus ; Pap smear, Papanicolaou smear test; ZAR, South African Rand.

References

1. **Medicines and related substances act (101 of 1965) regulations relating to a transparent pricing system for medicines and scheduled substances: Publication of the guidelines for pharmacoeconmic submissions** http://www.mediscor.net/docs/GG/Medicines%20and%20related%20Substances%20Act%20101-1965%20-%20Regulations%20relating%20to%20a%20transparent%20(20130201-GGR-36118-00068).pdf.

2. Department of Health, Republic of South Africa: **Uniform Patient Fee Schedule** http://www.health.gov.za/index.php/shortcodes/2015-03-29-10-42-47/2015-04-30-09-10-23/uniform-patient-fee-schedule.

3. **National Health Laboratory Service 2013** http://www.health.gov.za/docs/programes/2014/NHLS-StatePriceList2013ANNEXURE-M.pdf.

4. Goldie SJ, Kohli M, Grima D, Weinstein MC, Wright TC, Bosch FX, Franco E: **Projected clinical benefits and cost-effectiveness of a human papillomavirus 16/18 vaccine.** *J Natl Cancer Inst* 2004, **96:**604-615.

5. **South Africa Consumer Price Index (2008 - 2012)** http://www.statssa.gov.za/publications/P0141/CPIHistory.pdf?

6. International Monetary Fund: **World economic outlook database** http://www.imf.org/external/pubs/ft/weo/2013/01/weodata/index.aspx. Accessed: 9 May 2013

Delphi panel questionnaire
Health economic research project

Medical Management of HPV related disease

Dear Doctor,
Thank you for accepting to participate to our Delphi panel aiming at determining the medical management of patients with HPV related disease.
As you know, Cervarix®, a bivalent HPV vaccine, is to be available soon in South Africa. As part of the pricing and reimbursement submission for Cervarix®, a health economic analysis from the perspective of the South African health care system is to be provided to the authorities. The aim of such analysis is to determine the cost-effectiveness of introducing HPV vaccination compared to the current practice (screening) or between the two existing vaccines.
Given the delay between the expected age at vaccination (i.e. 12 years) and the age at which the expected benefit will be seen, such analysis is to be based on modelling. To populate the model data from different sources will be used. Data on effectiveness will be derived from clinical trials. The costs for all medical management used in the model i.e., cervical cancer screening, cervical dysplasia (CIN) treatment, warts treatment and cervical cancer treatment will be determined based on your input.
Therefore the analysis of resources used will be determined from expert opinion (two-round Delphi panel) based on the results of the current questionnaire to be independently filled in by a panel of 8 South African experts in charge of these patients in South Africa in a first round. The responses provided will thereafter be analysed (determination of average value) and presented to all of you for consolidation in a second and final round. The results on the final estimates will thereafter be included in the economic model.
This questionnaire is divided into 4 sections:

- Section 1 relates to the description of your specific expertise
- Section 2 deals with the description of the medical management of patients undergoing a cervical cancer screening in South Africa.
- Section 3 deals with the description of the medical management for CIN1, CIN2 and CIN3 and cervical cancer treatment
- Section 4 deals with the description of the medical management for genital warts treatment

Symptoms and hence medical management is likely to vary both within patients and between patients for each of the treatments investigated. For each, please try to consider all your patients (**helicopter view**) in the investigated situation (and not the individual patient). The management in this patient cohort may be considered to reflect different patient characteristics within each treatment pattern. We therefore ask you to estimate, in each section, the proportion of this patient cohort receiving each of the treatment options discussed.

For example, in Table 1 relating to the management of abnormal pap smears could be filled in as follows (**hypothetical numbers**):

**Diagnostic procedure following a positive pap smear**	**% of patients to whom this applies**
No additional procedure	**5**%
**1.5**: average number of Colposcopies	**20**%
**1**: average number of Colposcopies with biopsy	**2.5%**
**2.5**: average number of repeat pap smears	**100**%
**1**: average number of HPV tests	**10**%
**1**: Other, please specify: **thorax radiography**	**0.5**%
**….**: Other, please specify **……………………….**	**….**%

Section One: Expert Information

Please fill in hereunder some details about your area of expertise:

Name:

....

Field of expertise:

....

....

Medical practice

Please report in the table hereunder the number of patients attending your medical practice over a one year period for each reason:

	**Number of patients seen per year**
Cervical cancer screening	.... patients per year
Treatment of CIN1	.... patients per year
Treatment of CIN2	.... patients per year
Treatment of CIN3	.... patients per year
Treatment of cervical cancer	.... patients per year
Treatment of genital warts	.... men per year<br>.................. women per year
Number of patients treated with genital Chlamydia trachomatis	.... men per year<br>.................. women per year
Total number of patients in your practice	.... patients per year
Region covered by your practice	.... (name of region)
Population of the region	.... number of people

Section Two: Cervical cancer screening

The following section of the questionnaire refers to your current medical management of a patient with a positive PAP smear test. Please report hereunder all **diagnostic procedures** undergone by a patient following a positive pap smear (abnormal pap), as well as the proportion of patients to which each applies (please note that the sum of % can be higher than 100% due to multiple therapies):

Table 1: Medical management of a positive PAP smear

Diagnostic procedure following a positive pap smear	% of patients to whom this applies
No additional procedure	......%
......: average number of Colposcopies	......%
......: average number of Colposcopies with biopsy	......%
......: average number of repeat pap smears	......%
......: average number of HPV tests	......%
......: Other, please specify	......%
......: Other, please specify	......%

Section Three: CIN & cancer treatment

The following section of the questionnaire refers to medical management of patients following CIN1, CIN2, CIN3 or cervical cancer histology confirmed diagnosis. Please report hereunder, for each specific diagnostic (CIN1, CIN2 and CIN3), the average number of **physician visits** per year, the average number of **Pap smears per year**, all and number of **therapeutic procedures** undergone and the proportion of patients to which each applies (the sum of % can be higher than 100% due to multiple therapy and physician visits).

Table 2: Medical management of histology confirmed CIN1

	Resource use among all CIN1 patients	% of patients to whom this applies
**Physician visits**		
	......: Average number of GP visits per patient	......%
	......: Average number of gynaecologist visits per patient	......%
	......: Average number of other specialist visits per patient<br>Please specify.............................	......%
	......: Average number of pap smears per patient	......%
	......: Average number of days in hospital (in-patient, **normal room**)	......%
	......: Average number of days in hospital (in-patient, **intensive care unit**)	......%
	......: Average number of days in hospital (***day care***)	......%
**Treatment procedures**		
	None	......%
	......: Laser treatment	......%
	......: Cryotherapy	......%
	......: Conisation	......%
	......: Hysterectomy	......%
	......: Other, please specify	......%
	......: Other, please specify	......%

Table 3: Medical management of histology confirmed CIN2/3

	Resource use among all CIN2/3 patients	% of patients to whom this applies
**Physician visits**		
	......: Average number of GP visits per patient	......%
	......: Average number of gynaecologist visits per patient	......%
	......: Average number of other specialist visits per patient<br>Please specify.............................	......%
	......: Average number of pap smears per patient	......%
	......: Average number of days in hospital (in-patient, **normal room**)	......%
	......: Average number of days in hospital (in-patient, **intensive care unit**)	......%
	......: Average number of days in hospital (***day care***)	......%
**Treatment procedures**		
	......: None	......%
	......: Laser treatment	......%
	......: Cryotherapy	......%
	......: Conisation	......%
	......: Hysterectomy	......%
	......: Other, please specify	......%
	......: Other, please specify	......%

Table 4: Medical management of histology confirmed cervical cancer

	Resource use among all Cervical Cancer patients	% of patients to whom this applies
**Physician visits**		
	......: Average number of GP visits per patient	......%
	......: Average number of gynaecologist visits per patient	......%
	......: Average number of oncologist visits per patient	......%
	......: Average number of other specialist visits per patient<br>Please specify	......%
	......: Average number of other specialist visits per patient<br>Please specify..............................	......%
	......: Average number of other specialist visits per patient<br>Please specify..............................	......%
	......: Average number of other specialist visits per patient<br>Please specify..............................	......%
	......: Average number of pap smears per patient	......%
**Hospitalisations**		
	......: Average number of days in hospital (in-patient, ***normal room***)	......%
	......: Average number of days in hospital (in-patient, ***intensive care unit***)	......%
	......: Average number of days in hospital (***day care***)	......%

Table 4: **Medical management of histology confirmed cervical cancer (continued)**

	Resource use among all Cervical Cancer patients	% of patients to whom this applies
**Treatment procedures**		
	......: None	......%
	......: Laser treatment	......%
	......: Cryotherapy	......%
	......: Conisation	......%
	Hysterectomy	......%
	Radiotherapy<br>specify (dosage and number of sessions)<br>..<br>..<br>..	......%
	Chemotherapy<br>Specify regimen and number of cycles<br>..<br>..<br>..	......%
	......: Other, please specify	......%
	......: Other, please specify	......%
	......: Other, please specify	......%
	......: Other, please specify	......%

Section Four: Treatment of Genital warts

This section of the questionnaire refers to the medical management of patients with genital warts. Please report hereunder for all your patients with genital warts over a one year period, the average number of **physician visits**, all **pharmacological treatments** received and all **therapeutic procedures** undergone as well as the proportion of patients to which each applies, and, for the therapeutic procedures. Please note that the sum of % can be higher than 100% due to multiple treatment and physician visits.

Table 5: Medical management of women with genital warts (treatment and follow-up)

Resource use among all genital warts patients		% of patients to whom this applies
**Physician visits**		
	......: Average number of GP visits per patient per year	......%
	......: Average number of gynaecologist visits per patient per year	......%
	......: Average number of dermatologist visits per patient per year	......%
	......: Average number of other visits per patient per year; please specify	......%
**Pharmacological treatment**		
	Imiquimod<br>Dose per day ......<br>...... average number of days per year	......%
	Podophylotoxine<br>Dose per day ......<br>...... average number of days per year	......%
	Other, please specify:<br>................................<br>Dose per day ......<br>...... average number of days per year	......%
	Other, please specify:<br>................................<br>Dose per day ......<br>...... average number of days per year	......%
**Treatment procedures**		
	......: Average number of procedures in patients undergoing electrocoagulation	......%
	......: Average number of procedures in patients undergoing laser treatment	......%
	......: Average number of procedures in patients undergoing cryotherapy	......%
	......: Average number of procedures in patients undergoing Surgical excision	......%
	......: Average number of other procedures, please specify...............................	......%
	......: Average number of other procedures, please specify...............................	......%

THANK YOU FOR YOUR INPUT!!
