# Supplementary material for: Cost-effectiveness analysis of human papillomavirus vaccination in South Africa accounting for human immunodeficiency virus prevalence
Source: BMC Infect Dis. 2015 Dec 11;15:566. doi: 10.1186/s12879-015-1295-z (PMC4676856; doi:10.1186/s12879-015-1295-z)
Supplement: Additional file 3: — Input ranges for one way sensitivity analyses. (PDF 156 kb) [file 12879_2015_1295_MOESM3_ESM.pdf]

Additional file 3 – Input ranges for one way sensitivity analyses

Parameter	Base value	Minimum	Maximum
Oncogenic HPV incidence in women	0.041–0.390	0.033–0.312	0.050–0.467
HIV incidence rate	0.005–0.057	0.004–0.046	0.006–0.068
Mortality rate			
HIV-	0.001–0.182	0.001–0.146	0.001–0.218
HIV+	0.002–0.273	0.002–0.218	0.002–0.328
Disutility			
CIN1	0.0128	0.0102	0.0154
CIN2/3	0.0127	0.0102	0.0152
Cervical cancer	0.2730	0.2184	0.3276
Cervical cancer cured	0.062	0.496	0.0744
Baseline HIV+ utility	0.810	0.648	0.972
Screening coverage (%)	13.6	10.9	16.3
Screening start age (years)	30	24	36
Screening end age (years)	60	48	72
Screening interval (years)	3	2	4
Sensitivity pap screening			
CIN1	0.580	0.464	0.696
CIN2/3	0.610	0.488	0.732
Cost screening (ZAR)	256.00	205.00	307.00
Cost treatment (ZAR)			
CIN1	830.00	664.00	996.00
CIN2/3	2 464.00	1 971.00	2 957.00
Cervical cancer	40 507.00	32 406.00	48 608.00
Vaccine efficacy (%) [1]			
CIN1	50.3	40.2	58.8
CIN2/3	64.9	52.7	74.2
Cervical cancer	93.2	78.9	98.7

CIN, cervical intraepithelial neoplasia; CIN1, cervical intraepithelial neoplasia grade 1; CIN2/3, cervical intraepithelial neoplasia grade 2 or 3; HIV+, human immunodeficiency virus positive; HIV-, human immunodeficiency virus negative; HPV, human papillomavirus; ZAR, South African Rand.

1. Lehtinen M, Paavonen J, Wheeler CM, Jaisamrarn U, Garland SM, Castellsague X, Skinner SR, Apter D, Naud P, Salmeron J, Chow SN, Kitchener H, Teixeira JC, Hedrick J, Limson G, Szarewski A, Romanowski B, Aoki FY, Schwarz TF, Poppe WA, De Carvalho NS, Germar MJ, Peters K, Mindel A, De SP, Bosch FX, David MP, Descamps D, Struyf F, Dubin G: **Overall efficacy of HPV-16/18 AS04-adjuvanted vaccine against grade 3 or greater cervical intraepithelial neoplasia: 4-year end-of-study analysis of the randomised, double-blind PATRICIA trial.** *Lancet Oncol* 2012, **13:**89-99.

This is confidential information of the GSK group of companies. This document is only for review and should not be shared further. Premature disclosure of unpublished or unpresented material can result in refusal of publications by journals and congresses.
