# Supplementary material for: Cost-effectiveness analysis of human papillomavirus vaccination in South Africa accounting for human immunodeficiency virus prevalence
Source: BMC Infect Dis. 2015 Dec 11;15:566. doi: 10.1186/s12879-015-1295-z (PMC4676856; doi:10.1186/s12879-015-1295-z)
Supplement: Additional file 4: — Multivariate probabilistic sensitivity analysis input data. (PDF 64 kb) [file 12879_2015_1295_MOESM4_ESM.pdf]

Additional File 4 - Multivariate probabilistic sensitivity analysis input data

Variable names	Distribution	Distribution parameters	
		HIV- subpopulation	HIV+ subpopulation
**Transition probability**			
HPV-Onc to CIN1	Normal distribution between 0 and 1	0.049(SD. 0.009) [1]	0.096 (0.007) [2]
HPV-Onc clearance to normal	Uniform distribution	0.234-0.664 [1,3-5]	0.170-0.254 [2]
CIN1 to CIN2/3	Normal distribution between 0 and 1	0.091 (SD.0.021) [4-6]	0.098 (SD.0.013) [2]
CIN2/3 cured	Normal distribution between 0 and 1	0.227 (SD.0.058) [4-6]	Assumption (same as HIV- subpopulation)
CIN2/3 to persistent CIN2/3	Uniform distribution	0.091-0.137 [5,6]	Assumption (same as HIV- subpopulation)
Persistent CIN2/3 to cervical cancer	Uniform distribution	0-0.098	0-0.181 [7]
**Mortality rate**			
Age-dependent mortality data	Uniform distribution	0.001-0.064 [8]	0.001-0.449 [8]
**HPV and HIV incidence**			
HIV incidence rate in women	Uniform distribution	N/A	0.001-0.036 [8]
Oncogenic HPV incidence	Uniform distribution	0.033-0.468 [9]	0.088-1 [9] [10]
**Cervical cancer parameters**			
Cervical cancer mortality	Uniform distribution	0.088-0.132 [11]	0.233-0.349 [12]
Cervical cancer cured	Uniform distribution	0.121-0.181 [11]	0.022-0.033 [12]
**HPV screening-related parameters**			
CIN1 detected	Normal distribution between 0 and 1	0.58 (SD.0.045) [13]	0.58 (SD.0.045) [13]
CIN2/3 detected	Normal distribution between 0 and 1	0.61 (SD. 0.045) [13]	0.61 (SD. 0.045) [13]
**HPV-related disease management**			
Proportion of women treated if CIN1 is detected	Uniform distribution	0.4-0.6 (assumption)	Assumption (same as HIV- subpopulation)
CIN1 treatment success	Uniform distribution	0.72-1 (assumption)	Assumption (same as HIV- subpopulation)
CIN2/3 treatment success	Uniform distribution	0.72-1 (assumption)	Assumption (same as HIV- subpopulation)
**Vaccine efficacy [14]**			
Vaccine efficacy for CIN1+	Normal distribution between 0 and 1	0.503 (SD.0.054)	0.503 (SD.0.047)
Vaccine efficacy for CIN2/3	Normal distribution between 0 and 1	0.649 (SD.0.054)	0.649 (SD.0.054)
Vaccine efficacy for CC	Normal distribution between 0 and 1	0.932 (SD.0.050)	0.932 (SD.0.050)
**Disutility data [3,15-18]**			
No HPV (baseline value)	Fix at 0	N/A	N/A
HIV+ (baseline value)	Uniform distribution	N/A	0.648-0.972 [19]
HPV-Onc	Fix at 0	N/A	N/A
CIN1	Fix at 0	N/A	N/A

Variable names	Distribution	Distribution parameters	
		HIV- subpopulation	HIV+ subpopulation
CIN1 detected	Uniform distribution	0.010-0.015	0.010-0.015
CIN2/3	Fix at 1	N/A	N/A
CIN2/3 detected	Uniform distribution	0.010-0.015	0.010-0.015
Cervical cancer	Uniform distribution	0.218-0.328	0.218-0.328
Cervical cancer cured	Uniform distribution	0.050-0.074	0.050-0.074
**Cost data**			
Cost of regular screening negative Pap smear	Uniform distribution	ZAR 205-307	ZAR 205-307
Regular screening + false positive	Uniform distribution	ZAR 231-347	ZAR 231-347
Treatment CIN1 detected	Uniform distribution	ZAR 664-996	ZAR 664-996
Treatment CIN2/3 detected	Uniform distribution	ZAR 1 971-2 957	ZAR 1 971-2 957
Cervical cancer stage I-IV	Uniform distribution	ZAR 32 406-48 608	ZAR 32 406-48 608

Abbreviations

CC, cervical cancer; CIN, cervical intraepithaelial neoplasia; CIN1, CIN grade 1; CIN2/3, CIN grade 2 or 3; HPV, human papillomavirus; HPV-Onc, oncogenic HPV types; HIV, human immunodeficiency virus; HIV-, HIV-negative; HIV+, HIV+positive; Pap, Papanicolou smear test; SD, standard deviation; ZAR, South African Rand.

References

1. Moscicki AB, Hills N, Shiboski S, Powell K, Jay N, Hanson E, Miller S, Clayton L, Farhat S, Broering J, Darragh T, Palefsky J: **Risks for incident human papillomavirus infection and low-grade squamous intraepithelial lesion development in young females.** *JAMA* 2001, **285:**2995-3002.

2. Omar T, Schwartz S, Hanrahan C, Modisenyane T, Tshabangu N, Golub JE, McIntyre JA, Gray GE, Mohapi L, Martinson NA: **Progression and regression of premalignant cervical lesions in HIV-infected women from Soweto: a prospective cohort.** *AIDS* 2011, **25:**87-94.

3. Goldie SJ, Kohli M, Grima D, Weinstein MC, Wright TC, Bosch FX, Franco E: **Projected clinical benefits and cost-effectiveness of a human papillomavirus 16/18 vaccine.** *J Natl Cancer Inst* 2004, **96:**604-615.

4. Schlecht NF, Platt RW, Duarte-Franco E, Costa MC, Sobrinho JP, Prado JC, Ferenczy A, Rohan TE, Villa LL, Franco EL: **Human papillomavirus infection and time to progression and regression of cervical intraepithelial neoplasia.** *J Natl Cancer Inst* 2003, **95:**1336-1343.

5. Melnikow J, Nuovo J, Willan AR, Chan BK, Howell LP: **Natural history of cervical squamous intraepithelial lesions: a meta-analysis.** *Obstet Gynecol* 1998, **92:**727-735.

6. Holowaty P, Miller AB, Rohan T, To T: **Natural history of dysplasia of the uterine cervix.** *J Natl Cancer Inst* 1999, **91:**252-258.

7. De Vuyst H, Lillo F, Broutet N, Smith JS: **HIV, human papillomavirus, and cervical neoplasia and cancer in the era of highly active antiretroviral therapy.** *Eur J Cancer Prev* 2008, **17:**545-554.

8. Actuarial Society of South Africa: **ASSA2008: AIDS Demographic Model 2008** http://www.actuarialsociety.org.za/Societyactivities/CommitteeActivities/AidsCommittee/Models.aspx. Access granted in November 2012 and confirmed in November 2015 by the Actuarial Society of South Africa, owner of the model.

9. Institut Català d'Oncologia (ICO): **Information Centre on HPV and Cancer (HPV Information Centre)** http://www.hpvcentre.net/dataquery.php. Accessed: 10 Feb. 2014

10. Vijayaraghavan A, Efrusy M, Lindeque G, Dreyer G, Santas C: **Cost effectiveness of high-risk HPV DNA testing for cervical cancer screening in South Africa.** *Gynecol Oncol* 2009, **112:**377-383.

11. Ferlay, J., Soerjomataram, Isabelle, Ervik, M., Dikshit, R., Eser, S., Mathers, C., Rebelo, M., Parkin, D. M., Forman, D., and Bray F.: **GLOBOCAN 2012 v1.0, Cancer incidence and mortality worldwide: IARC CancerBase No. 11 [Internet]** http://globocan.iarc.fr. Accessed: 31 Jan. 2014 International Agency for Research on Cancer (IARC), Lyon, France

12. Coghill AE, Newcomb PA, Madeleine MM, Richardson BA, Mutyaba I, Okuku F, Phipps W, Wabinga H, Orem J, Casper C: **Contribution of HIV infection to mortality among cancer patients in Uganda.** *AIDS* 2013.

13. Fahey MT, Irwig L, Macaskill P: **Meta-analysis of Pap test accuracy.** *Am J Epidemiol* 1995, **141:**680-689.

14. Lehtinen M, Paavonen J, Wheeler CM, Jaisamrarn U, Garland SM, Castellsague X, Skinner SR, Apter D, Naud P, Salmeron J, Chow SN, Kitchener H, Teixeira JC, Hedrick J, Limson G, Szarewski A, Romanowski B, Aoki FY, Schwarz TF, Poppe WA, De Carvalho NS, Germar MJ, Peters K, Mindel A, De SP, Bosch FX, David MP, Descamps D, Struyf F, Dubin G: **Overall efficacy of HPV-16/18 AS04-adjuvanted vaccine against grade 3 or greater cervical intraepithelial neoplasia: 4-year end-of-study analysis of the randomised, double-blind PATRICIA trial.** *Lancet Oncol* 2012, **13:**89-99.

15. Gold MR, Franks P, McCoy KI, Fryback DG: **Toward consistency in cost-utility analyses: using national measures to create condition-specific values.** *Med Care* 1998, **36:**778-792.

16. Insinga R, Glass A, Rush B: **Health state transitions following an abnormal pap smear: implications for health utility assessment in cost-effectiveness analyses [Abstract W-02].** In *22nd International Papillomavirus Conference & Clinical Workshop 2005*; *30 Apr.-06 May 2005*.

17. Myers ER, Green S, Lipkus I: **Patient preferences for health states related to HPV infection: visual analog scale versus time trade-off elicitation [Abstract n° 542].** In *Twenty-First International Papillomavirus Conference*; *20-27 February 2004*.

18. Institute of Medicine (US) Committee to Study Priorities for Vaccine Development, Stratton KR DJLRe: **Appendix 11: Human Papillomavirus.** In *Vaccines for the 21st century: a tool for decisionmaking*. Edited by Edited by Stratton KR, Durch JS, Lawrence S. Washington D.C.: National Academy Press; 2000:213-221.

19. Robberstad B, Olsen JA: **The health related quality of life of people living with HIV/AIDS in sub-Saharan Africa - a literature review and focus group study.** *Cost Eff Resour Alloc* 2010, **8:**5.
